# Rural Household Differentiation and Poverty Vulnerability: An Empirical Analysis Based on the Field Survey in Hubei, China

**DOI:** 10.3390/ijerph19084878

**Published:** 2022-04-17

**Authors:** Zhengjie Zhang, Jiahao Song, Caixia Yan, Dingde Xu, Wei Wang

**Affiliations:** 1 Department of Marketing, School of Business and Tourism, Sichuan Agricultural University, 211 Huimin Rd, Chengdu 130062, China; hnsc1016@sicau.edu.cn; 2 Department of Rural and Regional Development, College of Management, Sichuan Agricultural University, 211 Huimin Rd, Chengdu 130062, China; 14741@sicau.edu.cn; 3 Sichuan Center for Rural Development Research, College of Management, Sichuan Agricultural University, 211 Huimin Rd, Chengdu 130062, China; yancx-cnd@sicau.edu.cn; 4 Department of Agriculture and Forestry Economics and Management, College of Management, Sichuan Agricultural University, 211 Huimin Rd, Chengdu 130062, China

**Keywords:** farm household differentiation, farmers’ livelihoods are sustainable, poverty vulnerability, regression decomposition method

## Abstract

Rural family differentiation is an important perspective to analyze farmers’ behavior and poverty. Based on the data of 1673 farm households from rural field survey in 2019 in Hubei Province of China, this paper examines the main influencing factors of farm household differentiation on farm household poverty vulnerability from the perspective of the sustainable livelihoods of farm households. On this basis, the contribution of each influencing factor to farm household poverty vulnerability is analysed using the regression decomposition method. The results of the study show that the variables of farm household differentiation have a significant impact on poverty vulnerability, and the net household income per capita, which reflect the vertical differentiation of farm households, and the proportion of non-farm labor, which reflects the horizontal differentiation of farm households. Both have a significant negative impact on the poverty vulnerability of farm households. The regression decomposition method shows that the proportion of non-farm labor force, which reflects the horizontal differentiation of farm households, has the highest contribution to the poverty vulnerability of farm households. Human capital, natural capital, social capital, and physical capital also influence the poverty vulnerability of farm households to a certain extent.

## 1. Introduction

Reducing poverty is one of the UN Millennium Goals and an important task facing developing countries [1,2,3,4]. As the world’s largest developing country, China has made great achievements in poverty reduction, with the incidence of poverty in China falling from over 60% in 1990 to less than 30% in 2002, thereby halving the proportion of people living in poverty and achieving full poverty eradication by 2020 [5,6]. The primary task of poverty alleviation is to accurately understand the poverty status of the poor and thus implement targeted measures to reduce and alleviate poverty [7]. However, the standard poverty measure is a static measure of a household’s welfare at a specific point in time and does not take into account the future welfare of the household or the risks associated with that welfare but is instead an ex-post measure [8,9]. While the standard poverty measure reflects the poverty status of the poor to a certain extent, this measure is unable to predict the future poverty status of the poor [8]. Moreover, the anti-poverty policies formulated on this basis are limited, thus increasing the likelihood that those who have escaped poverty, as well as those who are not already poor, will fall into poverty [10].

At present, the regional problems of unbalanced and inadequate development in China remain prominent [11,12,13]. Further, most of the areas that have been recently lifted out of poverty are deeply impoverished areas [14], with common characteristics such as deep poverty, weak development foundations, and an insufficient endogenous driving force, as well as significant vulnerability to poverty [15,16]. In addition, as the instability of the internal and external environment of China’s economic and social development further intensifies, the consolidation and maintenance of China’s poverty eradication achievements are facing great risks and challenges [11,13,17]. Based on this reality, the present study proposes to use data from a rural household survey in Hubei Province, China, to explore the mitigation of poverty vulnerability and sustainable livelihood formation among farm households from the perspective of household differentiation. The possible marginal contributions of this paper are as follows. First, to explore poverty vulnerability alleviation and the sustainable livelihood formation of farm households from the perspective of farm household differentiation based on Amartya Kumar Sen’s capability rights system framework and the development reality of rural areas in Hubei Province, China; and second, to explore the poverty vulnerability alleviation of farm households using household research data in 2019 from the decisive period of poverty eradication. The second objective is to explore the mechanisms of poverty vulnerability alleviation for rural households. The results will provide a reference for relevant decision making and an empirical basis for consolidating the poverty reduction achievements and achieving sustainable development in China.

The remainder of the paper is organized as follows. Part 2 contains a logical analysis of the relevant theories. Part 3 contains the data sources, the selection of variables, and the descriptions of the empirical model. Parts 4 and 5 are the main parts of the paper, which measure poverty vulnerability. On this basis, the net income per capita of farm households and the proportion of non-farm labor force are used as indicators for the horizontal and vertical differentiation of farm households, respectively, to explore the impact of the poverty vulnerability of farm households. Part 6 contains the results which were obtained from the empirical model and attempts to explain the underlying mechanisms. Part 7 discusses the innovations and limitations and puts forward policy implications.

## 2. Theoretical Analysis

### 2.1. Poverty Vulnerability and Sustainable Livelihoods

With the development of anti-poverty theory, scholars have gradually shifted their focus from observing poverty to predicting poverty and exploring poverty vulnerability, which is caused by factors such as uncertainty of future household income and risk [7,10,18]. The World Bank first introduced the concept of ‘poverty vulnerability’ in 2000 as an indicator for the likelihood of a farm household falling into poverty [19,20,21,22]. In contrast to traditional poverty measures of income or consumption thresholds, poverty vulnerability can be used to predict the future poverty status of farm households and formulate proactive anti-poverty policies [23]. This forward-looking concept provides a new perspective on the study of poverty and is gradually becoming a hot topic of research in the field of farm household livelihoods [12,24,25,26]. At present, there is no uniform definition of poverty vulnerability in academic circles, and different methods for measuring poverty have been proposed based on different definitions [27]. The method most widely accepted by international scholars is Vulnerability as Expected Poverty (VEP) proposed by Chaudhuri, which emphasizes that VEP is a forward-looking projection of a household’s future welfare status [28]. Literature studies based on the theoretical approach of Chaudhuri have focused on two main areas, one of which is the study of poverty vulnerability measures [28]. Azeem et al. estimated poverty vulnerability in the Punjab province of Pakistan through consumption expenditures and food calorie intake [29]. Ozughalu and Uche studied the relationship between household food poverty and food poverty vulnerability in Nigeria and calculated region-specific food poverty lines based on the food types common to each geopolitical zone [30]. The second method is the analysis of factors influencing vulnerability. Sohns et al. found that the poverty vulnerability of farm households with bank credit was 0.4% lower than the poverty vulnerability among those without bank credit after studying the correlation between credit access and poverty vulnerability [31]. Han et al. studied the impact of inclusive financial policies on farm poverty vulnerability in China and found that the spread of inclusive financial policies could significantly reduce farm poverty vulnerability [32]. Through a study of tea farmers’ livelihood levels in Bangladesh, Islam found that low living standards were the most important factor leading to poverty vulnerability among tea farmers [33].

Since 1992, when the United Nations Conference on Environment and Development introduced the concept of ‘livelihoods’ into the action agenda and advocated the achievement of stable livelihoods as the main objective of poverty eradication, the concept of ‘livelihoods’ has become an important consideration for researchers studying rural economies and farm poverty [34,35,36,37]. Building on Sen’s theoretical work on the nature of poverty, the UK Department for International Development (DFID) developed a framework for sustainable livelihood analysis in 1998 [38]. In this theoretical framework, the sustainable livelihoods of farm households consist of five different livelihood capitals, with a coupling relationship between each livelihood capital. When one livelihood capital of a farm household is missing, that capital can be maintained in a relatively stable pentagonal state through a complementary relationship with other livelihood capitals, thus achieving the sustainable development of farm household livelihoods. Mthethwa and Wale used this theoretical framework to study the poverty vulnerability of farm households in South Africa and found that different livelihood capitals have different impacts on farm poverty vulnerability, with human and financial capital playing a key role in making rural households resilient to food insecurity vulnerability [39]. Sayema et al. used this theoretical framework and found that poverty vulnerability is more pronounced among rural scavengers in Bangladesh, while also calling for local government attention to the issue [40]. Through a study of climate-sensitive areas in China, Ward found that differences in social capital led to significant differences in farm household poverty vulnerability [41].

Overall, scholars can use this framework to link the many factors affecting poverty vulnerability to livelihood capital and clarify the multiplicity of interactions between different factors.

### 2.2. Mechanism of Poverty Alleviation and Sustainable Livelihood Formation Based on the Perspective of Farm Household Differentiation

Farm household differentiation is an important perspective in the study of farm household poverty [42]. With the continuous development of urbanization and industrialization in China, as well as the rapid progress of agricultural industrial restructuring and rural market reforms, the differences between farming households in levels of agricultural production technology and their ability to participate in the market continue to widen [43]. As a result, there is an increasingly clear division between traditional and modern farmers and significant differences in the ownership of livelihood capital between different types of farmers [44,45,46].

**Figure 1:**
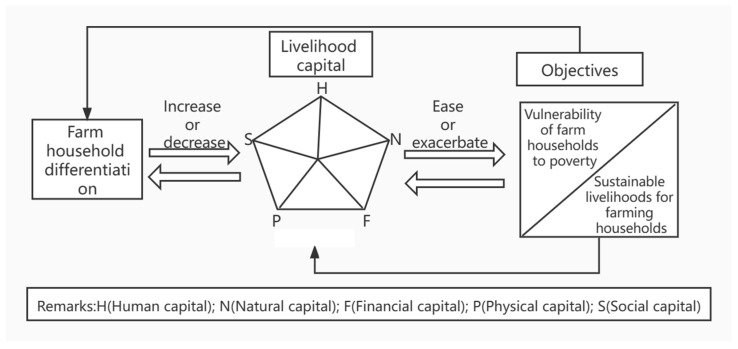
Mechanisms of farm household differentiation, poverty vulnerability, and sustainable livelihood formation.

The impact of farm household differentiation on the livelihood capital of farm households is mainly manifested as a widening gap in farm household income and differences in labor allocation. On the one hand, the production behavior of farm households has changed from homogeneous agricultural production to heterogeneous non-farm and part-time business, and the differences in business practices have led to the differentiation of farm household incomes, widening farm household income inequality and giving farm households at the bottom levels of the income differentiation a weaker ability to resist poverty risk shocks. These factors make such households more likely to fall into poverty when subjected to adverse risk shocks, resulting in unsustainable livelihoods [47,48,49]. On the other hand, the advancement of industrialization and urbanization has prompted a large number of rural laborers to shift from the agricultural sector to the non-agricultural sector, resulting in changes to the allocation of labor among non-agricultural laborers and a corresponding change in the income structure, with diversified sources of income effectively preventing farmers from falling into poverty and thus achieving sustainable livelihoods [50,51] (see Figure 1). However, the above represents only a theoretical analysis of the relationship between household differentiation, poverty vulnerability, and sustainable livelihood development. To better explore the pathways of sustainable livelihood development and clarify the extent to which different factors affect poverty vulnerability, an empirical analysis based on household differentiation is needed.

## 3. Data, Variable, and Model

### 3.1. Data

During July to August 2019, more than ten members of the subject group went to Huanggang City, Jingzhou City, Xiangyang City, and Huangshi City in Hubei Province to conduct field research. The research was conducted using stratified random sampling, with Shacheng District, Laohekou City, Yingshan County, Herb Spring County, and Yangxin County selected as sample counties (districts and cities) according to their levels of economic development. To conduct the survey, 12 villages were randomly selected from each county (district and city), and 30 households were randomly selected from each village. A total of 1800 questionnaires were distributed, and 1765 households were returned. After excluding invalid samples and missing samples of key variables, a valid sample of 1673 households was obtained, accounting for 94.79% of the total sample. The contents of the questionnaire (see Supplementary Materials: Table S1) included the basic information on farming households, mainly the age, gender, education level, type of work, health status, and insurance participation of household members; the assets of farming households, mainly production assets and living assets; the production and operation of farming households, mainly the income, cost, and other types of income from farming and breeding; the consumption situation of farming households, mainly living expenses, education expenses, medical expenses, and other expenses; and the social resources of farming households, mainly the social interaction situation of farming households.

### 3.2. Variable

Here, the dependent variable is the degree of poverty vulnerability among farm households, which was further refined by Bronfman based on the poverty vulnerability measure proposed by Chaudhuri [28,52] and estimated as follows:

(1) $$V_{i}{}_{t}=P_{r}(Y_{i}{{}_{,t}}_{+1}\leq Z)$$

where $Y_{i,t+1}$ denotes the per capita income level of farm household i at moment t+1, Z is the poverty line, and $V_{i}{}_{t}$ denotes the poverty vulnerability value of farm household i at moment t+1. This reflects the probability that the per capita income level of farm household i at moment t+1 will be below the poverty line Z.

It is generally accepted that the log-normal distribution is more suitable for describing the income levels of low-income farmers, so it is assumed that the income level of farmers obeys a log-normal distribution. Therefore, it is necessary to estimate the future income levels of farmers. The expression of future income level is as follows:

(2) $$LnY_{i}=f(X_{i},H_{i},\varepsilon_{i})=\beta_{i}X_{i}+{\beta^{\prime }}_{i}H_{i}+\varepsilon_{i}$$

where $X_{i}$ and $H_{i}$ denote the individual and household characteristics of farm households, respectively; $\beta_{i}$ and $\beta_{i}{}^{\prime }$denote the estimated coefficients of $X_{i}$ and $H_{i}$, respectively; and $\varepsilon_{i}$ is a disturbance term with a mean of zero, which represents a heterogeneous shock to income or consumption. Here, it is assumed that in a relatively stable economic structure, future changes to income or consumption are caused by heterogeneous shocks to $\varepsilon_{i}$. The unbiased estimation of $\varepsilon_{i}$ presupposes that the income or consumption $LnY_{i}$ of the farm household is homoskedastic, but this is not possible in reality. Thus, we assume that the variance of $\varepsilon_{i}$ depends on the following equation:

(3) $$Ln\delta_{\varepsilon ,i}^{2}=\lambda_{i}X_{i}+{\lambda^{\prime }}_{i}H_{i}$$

The use of ordinary squares parameter estimation results in biased estimates due to the presence of heteroskedasticity. To solve this issue, we draw from the method of Chaudhuri [28]. Using three-stage feasible generalized least squares (FGLS), the estimation of Equations (2) and (3) yields $\beta_{i}$, $\beta_{i}{}^{\prime }$, $\lambda_{i}$, and $\lambda_{i}{}^{\prime }$, which, in turn, allows for the expected consumption or income of farm household and its variance:

(4) $$\hat{E}[LnY_{i}|X_{i},H_{i}]={\hat{\lambda }}_{i}X_{i}+{{\hat{\lambda }}^{\prime }}_{i}H_{i}$$

(5) $$\hat{V}[LnY_{i}|X_{i},H_{i}]=\delta_{\varepsilon ,i}^{2}={\hat{\beta }}_{i}X_{i}+{\hat{\beta }}_{i}{}^{\prime }H_{i}.$$

Assuming that the logarithm of consumption or income follows a normal distribution, the vulnerability to poverty can be derived from the following equation:

(6) $$\begin{array}{cc} V_{it} & =P_{r}(Y_{i}{{}_{,t}}_{+1}\leq Z|X_{i},M_{i})=\phi [\frac{LnZ-LnY}{\sqrt{{\hat{\delta }}_{\varepsilon ,i}^{2}}}]\\ & =\phi [\frac{LnZ-({\hat{\beta }}_{i}X_{i}+\beta_{i}{}^{\prime }H_{i})}{\sqrt{{\hat{\lambda }}_{i}X_{i}+{{\hat{\lambda }}^{\prime }}_{i}H_{i}}}] \end{array}$$

where ϕ(·) is a normal distribution function, and the calculated probability value $V_{it}$ is the poverty vulnerability value. In the measurement process, the national poverty line and the World Bank poverty line were used to select the poverty thresholds. Here, the national poverty line is taken as the 2011 poverty standard constant price of USD $1.25, and the World Bank poverty line is selected as USD $1.9.

Independent variables include key variable and control variables. The focus of this paper is the impact of farm household differentiation on poverty vulnerability. Therefore, the degree of farm household differentiation is the key variable. Most scholars classify farm household differentiation into horizontal and vertical differentiation [53,54,55], where horizontal differentiation is measured by the proportion of non-farm labor in the household labor force, reflecting the degree of occupational differentiation of farm households, and vertical differentiation is measured by the net household income per capita, reflecting the degree of economic differentiation of farm households. At the same time, we also introduce characteristic variables for household head and livelihood capital as control variables in this paper, including human capital variables, natural capital variables, material capital variables, and social capital variables. It should be noted that financial capital variables are not classified separately as they overlap with the farm household differentiation variables. The household head characteristic variables include the age, gender, health status, and education level of the household head. The human capital variables include the household size, population burden coefficient, and proportion of trained labor force. The natural capital variable is the land area per capita, the social capital variable is the number of relatives to and from the household, and the physical capital variable is the number of productive assets. Table 1 gives the definition, descriptive statistics, and expected influence direction of each variable. Due to space limitations, we will not repeat these elements.

### 3.3. Model

Using the poverty vulnerability values measured by the above method as the explanatory variables and the farm household differentiation variables, the head of the household characteristics and household characteristics were selected in this paper as explanatory variables. A multiple linear regression model was then constructed as follows:

(7) $$\begin{array}{cc} \;Vul_{i} & =\alpha +\beta_{1}income_{i}+\beta_{2}non\_agri_{i}+\beta_{3}gender_{i}+\beta_{4}age_{i}\\ & +\beta_{5}health_{i}+\beta_{6}edu_{i}+\beta_{7}size_{i}+\beta_{8}dep\_rate_{i}+\beta_{9}tra\_rate_{i}\\ & +\beta_{10}land_{i}+\beta_{11}asset_{i}+\beta_{12}relative_{i}+\varepsilon_{i} \end{array}$$

where $Vul_{i}$ denotes the farm household poverty vulnerability value, α denotes the constant term, $\beta_{i}$ is the coefficient to be estimated, and $\varepsilon_{i}$ is the residual term.

## 4. Results

### 4.1. Model Estimation Results

We measured the poverty vulnerability of farm households using Stata 13.0 and, based on the results, estimated each parameter of model Equation (7) (see Equation (1) in (Table 2)). We found that all variables were significant above a 10% level, except for the years of education of the household head and the area of land operated per capita. Moreover, the signs of the estimated coefficients of all variables were consistent with expectations, except for the area of land operated per capita.

The farm household differentiation variables were shown to have a significant effect on the vulnerability of farm households to poverty. Specifically, both net household income per capita and the proportion of non-farm labor have a significant negative effect on the poverty vulnerability of farm households. The higher the per capita net household income is, the lower the poverty vulnerability of farming households becomes. Conversely, increasing household income can effectively reduce the risk of falling into poverty. On the other hand, the higher the proportion of non-farm labor is in farm households, the lower the poverty vulnerability of farm households and the stronger the sustainable livelihood capacity of farm households are. In terms of household head characteristics, households headed by men were found to be at lower risk of falling into poverty. The statistical results show that when the head of the household is male, the poverty vulnerability value is 0.36; however, when the head of the household is female, the poverty vulnerability value is 0.54. The age of the head of the household also has a significant positive effect on the poverty vulnerability of the farming household: the older the head of the household is, the greater the risk of the farming household falling into poverty. The health status of the head of the household has a significant negative effect on the poverty vulnerability of the farming household, where the risk of the farming household falling into poverty is greater if the head of the household is unhealthy. The health status of the household head has a significant negative effect on the vulnerability to poverty among farming households. In terms of human capital variables, the larger the household size and the higher the population burden coefficient are, the greater the risk of falling into poverty, while training in professional skills among the household labor force can significantly reduce the vulnerability of farming households to poverty and enhance the ability of households to engage in sustainable livelihoods. The effect of natural capital on household poverty vulnerability was found to be not significant.

### 4.2. Robustness Tests

To test the robustness of the above estimation results, the parameters must be re-estimated by rounding off some variables and choosing different poverty criteria (see Table 2). Equation (2) removes two variables (the years of education of the household head and the size of the household) from Equation (1); and Equation (3) removes two variables (the training status of professional skills and the area of land per capita) from Equation (1). Equation (4) is the poverty vulnerability value calculated by the World Bank’s USD $1.25 poverty line, and Equation (5) is the poverty vulnerability value calculated by the World Bank’s USD $1.9 poverty line. Table 2 clearly indicates that the magnitude, direction, and significance of the coefficients for the farm household differentiation variables and other control variables of interest in this paper remained largely unchanged, so the estimation results of Equation (1) are robust.

## 5. Impact Extent of Farm Household Differentiation on Poverty Vulnerability

The estimated results of the model parameters presented in Table 2 only reflect whether different variables have an impact on farm household poverty vulnerability. The exact contribution of farm household differentiation variables, household head characteristics, and household characteristic variables to the impact of farm household poverty vulnerability, as well as the size of the effect of farm household differentiation, need to be further measured. Based on the estimation results of Equation (1), the contribution rate of each variable to the impact of farm household poverty vulnerability is measured using Wan’s improved regression decomposition method [56,57]. The specific method involves simplifying Equation (7) as follows:

(8) $$Vul_{i}=\alpha +\beta_{i}X_{i}+\varepsilon_{i}$$

where $Vul_{i}$ denotes the poverty vulnerability value of the farm household i, α denotes the constant term, $X_{i}$ denotes the explanatory variable, and $\varepsilon_{i}$ denotes the residual term. After estimating the parameters of Equation (8), an estimate of $\beta_{i}$ is obtained, and then an estimate of $Vul_{i}$, ${\hat{V}}_{i}$ is calculated, as well as an estimate of ${\hat{V}}_{i}{}^{\prime }$, $Vul_{i}$ when the constant term is not considered:

(9) $${\hat{V}}_{i}=\alpha +\sum_{i=1}^{k}\beta_{i}X_{i}$$

(10) $${\hat{V}}_{i}{}^{\prime }=\sum_{i=1}^{k}\beta_{i}X_{i}$$

The regression decomposition is then performed. In step 1, denote the coefficient of variation using CV(*) and calculate the contribution of the residual and constant terms to $Vul_{i}$. $C_{\varepsilon }$ and $C_{\alpha }$:

(11) $$C_{\varepsilon }=CV(V)-CV(\hat{V})$$

(12) $$C_{\alpha }=CV(\hat{V})-CV({\hat{V}}^{\prime })$$

Then in step 2, alculate the contribution of each variable to $CV(\hat{V})$. Using the Shapley value theory decomposition proposed by Shorrocks the contribution of each variable to $CV(\hat{V})$ can be calculated [58]. In general, different farmers take different values of X. Replacing X with the sample mean of $X_{i}$ eliminates the variance of $X_{i}$. The value of V calculated after the replacement is noted as ${\hat{V}}_{i}$ (without the constant term), which gives the coefficient of variation $CV({\hat{V}}_{i})$. Here, $CV({\hat{V}}_{i})$ depends on the variability of X after the elimination of $X_{i}$. Similarly, replacing $X_{i}$ and $X_{j}$ with the sample means of $X_{i}$ and $X_{j}$ eliminates the variance of $X_{i}$ and $X_{j}$. The value of V calculated after the replacement is noted as ${\hat{V}}_{i}$, which gives the coefficient of variation $CV({\hat{V}}_{ij})$. By continuing this process, more variation in X can be eliminated.

Denoted by $C_{i}^{mn}$, the contribution of variable i to the coefficient of variation in the nth of the mth value is calculated as follows:

(13) $$C_{i}^{1n}=CV({\hat{V}}^{\prime })-CV({\hat{V}}_{i})\;i=1,2,\cdots ,k$$

(14) $$C_{i}^{2n}=CV({\hat{V}}_{i}{}^{\prime })-CV({\hat{V}}_{ij})\;i,j=1,2,\cdots ,k(i\neq j)$$

(15) $$C_{i}^{3n}=CV({\hat{V}}_{ij}{}^{\prime })-CV({\hat{V}}_{ijp})\;i,j,p=1,2,\cdots ,k(i\neq j\neq p)$$

The contribution of variable i to the coefficient of variation at time m is

(16) $$C_{i}^{m}=\sum_{m=1}^{N_{m}}C_{i}^{m}/N_{m}$$

(17) $$N_{m}=\frac{(k-1)!}{(k-m)!(m-1)!}$$

where $C_{i}^{m}$ denotes the contribution of variable i to the coefficient of variation at time m. The contribution of variable i to the variation in poverty vulnerability among farm households is

(18) $$C_{i}=\sum_{m=1}^{k}C_{i}^{m}/k$$

At last, in step 3, easure the contribution of each variable to the vulnerability of farm households:

(19) $$CD_{\varepsilon }=\frac{C_{\varepsilon }}{CV(V)}\times 100\%$$

(20) $$CD_{\alpha }=\frac{C_{\alpha }}{CV(V)}\times 100\%$$

(21) $$CD_{i}=\frac{C_{i}}{CV(V)}\times 100\%$$

where $CD_{\varepsilon }$, $CD_{\varepsilon }$, and $CD_{\varepsilon }$ denote the residual term, the constant term, and the contribution of each variable to the difference in poverty vulnerability of farm households, respectively.

Table 3 gives the contribution of the residual, constant, and explanatory variables to poverty vulnerability. Although the regression decomposition method cannot identify the contribution of explanatory variables that are not included, these unidentified variables are reflected in the residual terms. Here, we measured the contribution of the residual term to farm poverty vulnerability at 28.24% and the contribution of the explanatory variables to farm poverty vulnerability at 78.58%. Thus, even if there are unobservable factors not included in the regression model, the explanatory variables selected in this paper can still explain, to a large extent, the impact on the poverty vulnerability of farm households.

The regression decomposition method was further used to measure the contribution of each explanatory variable to the poverty vulnerability of farm households, and the results are shown in Table 4. The results clearly show that the proportion of non-farm labor force has the largest contribution to the poverty vulnerability of farm households, reaching 35.05%. Thus, the horizontal differentiation of farm households has the greatest impact on the poverty vulnerability of farm households; while the vertical differentiation of farm households has a relatively small impact on the poverty vulnerability of farm households, with a contribution rate of only 1.49%. In addition to the variables of farm household differentiation, the contributions of household size, population burden coefficient, status of receiving professional skills training, and the number of productive assets to the poverty vulnerability of farm households all exceeded 10%, while the contributions of the gender of the household head, the health of the household head, the number of years of education of the household head, the area of land operated per capita, and the number of close relatives to the poverty vulnerability of farm households were all lower (below 5%).

## 6. Discussion

Many scholars have conducted in-depth research on poverty vulnerability, how to reduce the possibility of farmers falling into poverty in the future is a hot topic in many current studies. Compared with the existing studies, this study mainly makes the following three contributions. Firstly, this paper studies poverty vulnerability from the perspective of household differentiation, combing the literature, we found that few pieces of literature carry out research from this perspective. Secondly, this paper constructs the analysis framework of rural household differentiation and potential vulnerability, the mechanism of poverty vulnerability mitigation and sustainable livelihood formation from the perspective of farmers’ differentiation, and measures farmers’ poverty vulnerability. Thirdly, this paper empirically analyzes the impact of farmers’ differentiation on poverty vulnerability by using micro survey data and discusses the impact of different factors on Farmers’ poverty vulnerability by using regression decomposition method. This study aims to explore how to optimize farmers’ coping behavior from the perspective of farmers’ differentiation. At the same time, the differentiation of farmers is divided into horizontal differentiation and vertical differentiation, and further explores the determinants of farmers’ poverty vulnerability.

Although we sought to refine our research as much as possible, this study still inevitably has some shortcomings. First, the limited number of years of cross-sectional data we used may have had a negative impact on our empirical results. In the future, we plan to continue our study with richer panel data. Second, in order to make our study more detailed, we need to further expand the dimension of household differentiation, which is our future research direction.

## 7. Conclusions and Policy Implications

### 7.1. Conclusions

The results of the baseline regression model show that the variable of household differentiation has a significant negative effect on the vulnerability to poverty among farming households. The higher the per capita net income of a farm household is, the lower the value of the poverty vulnerability of the farm household becomes. Thus, a higher income can effectively reduce the risk of a household falling into poverty, likely because the per capita net income reflects the household’s business and development capacity in the current year, which most directly reflects the farm household’s livelihood capacity. For rural households with higher incomes, in the absence of large external risk shocks, the net income per capita in the following year is less volatile; even if such households suffer a severe risk shock, they can use their accumulated savings to mitigate that risk shock. Thus, the higher the net income per capita is, the less likely it becomes that the household will fall into poverty [5,6]. Moreover, the higher the proportion of non-farm labor is, the lower the vulnerability of farm households to poverty becomes [14]. On the one hand, households engaged in non-farm activities have more diversified sources of income and a richer supply of various types of livelihood capital, making such households more resilient to poverty risk shocks; on the other hand, as agricultural production is constrained by natural resources and the market for agricultural products, such households are more likely to be vulnerable to poverty [18]. On the other hand, agricultural production is subject to both natural resource constraints and agricultural markets, which makes agriculture riskier and farm income relatively more volatile [31,33]. Farmers in non-farm employment, on the other hand, are less vulnerable to poverty because they only partially bear the risks of agricultural production (or bear none at all). This phenomenon is confirmed by the results of the descriptive statistics, in which the magnitude of farm household poverty vulnerability values are 0.58, 0.48, 0.31, and 0.13 when the proportion of household non-farm labor is below 0.25, 0.25 to 0.5, 0.5 to 0.75, and above 0.75, respectively.

Looking at the characteristics of the household head, the results may be due to the fact that when the household is headed by a male, the household has more labor resources and can choose from a variety of livelihood options, thus reducing the likelihood of the household falling into poverty and achieving sustainable livelihood development. The age of the household head also has a significant positive effect on the poverty vulnerability of the farming household. However, the health status of the household head has a significant negative effect on the poverty vulnerability of farming households [23,29], likely because the health status of the household head, as the key labor force of the household, has a greater impact on the ability of the whole household to generate income, thus leading to differences in poverty vulnerability. In terms of human capital, the larger the household size and the higher the population burden coefficient are, the more likely the household is to fall into poverty [30,31]. There are two possible reasons for this result. First, when the number of non-working individuals (elderly or children) in the household is higher, the burden of supporting the household labor force is greater, thus leading to a reduction in the household’s ability to withstand risk and a greater vulnerability to fall into poverty. The second factor is that the family workforce may be squeezed out of their labor time or energy by caring for the non-workforce members of the household [9,26], creating lost opportunities to generate income for the family and leading to poverty. In contrast, professional skills training can enhance the work capacity of the household workforce and contribute to the sustainability of household livelihoods. In terms of social capital, households that possess, and are in close contact with, a greater number of relatives are at a significantly lower risk of falling into poverty [41,59] because the vulnerability of farm households to poverty is influenced by a number of uncertainties, and the impacts of these factors on the farm economy rely heavily on access to information and the allocation of resources. Social capital acts as an informal mechanism to enhance the ability of farm households to achieve sustainable livelihoods through timely access to information and resources, linked by blood, geo, and kinship ties [52]. In terms of physical capital, it seems contrary to common sense that productive fixed assets will increase the likelihood of households falling into poverty. However, after analysis, this assertion appears justified because productive fixed assets generally refer to durable goods used in household agricultural production and are not directly equivalent to livelihood capital at the household’s disposal. Instead, the purchase of these large durable goods leads to a reduction in the household’s resilience to risk and, therefore, to poverty.

The proportion of non-farm labor contributes the most to the poverty vulnerability of farm households—i.e., the horizontal differentiation of farm households has the greatest impact on the poverty vulnerability among those households [55,56], whereas the vertical differentiation of farm households has a relatively smaller impact on poverty vulnerability. This result may be due to the fact that the horizontal differentiation of farm households has led to the gradual separation of farm households from agricultural production, as increasingly more farm households have started to change from pure agricultural production to part-time production, and some have given up agricultural production altogether and become non-farm production households. The horizontal differentiation of farming households is mainly manifested in two aspects. The first aspect involves part-time farming or non-farm business. Diversified production and management behavior can spread or transfer agricultural risks, as well as overcome various problems such as insufficient capital investment in agricultural production and imperfect markets for agricultural products [18]. Second, rural laborers work outside the home. Compared to income from agricultural production and management, income from working outside the home is usually higher and more stable. Both of these factors can increase the ability of farming households to resist risks and thus reduce their vulnerability to poverty [60,61]. The vertical differentiation of farm households may contribute less to the vulnerability of farm households to poverty because the vulnerability of farm households to poverty reflects the likelihood of farm households falling into poverty in the future, whereas current income does not reflect the ability of farm households to resist risks in the future.

### 7.2. Policy Implications

Based on the findings of this paper, the level of horizontal differentiation among farm households should be increased to reduce the vulnerability of such households to poverty. The horizontal differentiation of farm households is mainly reflected in the transfer of non-farm labor. Therefore, the proportion of non-farm labor among farm households can be increased through various channels to promote the horizontal differentiation of farm households and thus to reduce the vulnerability of farm households to poverty. First, we can promote the orderly transfer of rural land through various forms and standardize the procedures as well as improve the market, for the transfer of rural land. On the one hand, land transfer can greatly promote the large-scale operation of agricultural production and can enhance the efficiency of agricultural operations; on the other hand, land transfer can also help the labor force of farmers who have left the land to obtain higher and more stable incomes by working and engaging in non-agricultural industries. Second, targeted training of farmers’ professional skills will increase the adaptability of farmers to non-agricultural activities and increase the proportion of non-agricultural labor among farmers, thereby effectively reducing the likelihood of farmers falling into poverty in the future. Third, the labor market should be continuously improved to provide specialized information services for rural laborers, build a unified urban and rural employment system, and protect the rights and interests of transferred rural laborers in order to enhance their income stability. Fourth, we should further integrate urban and rural public services and provide various guarantees for both farmers engaged in non-farm business and farmers working outside the country to promote the horizontal differentiation of farmers and to improve their abilities to cope with poverty risks.

## Figures and Tables

**Table 1:** Variable definitions, descriptive statistics, and expected direction of impact.

Variable	Variable Name	Variable Code	Meaning of Variables	Mean	Variance	Expected Direction
Explained variable	Vulnerability value of farm poverty	vul	Measured results	0.3258 ^1^	0.3605	—
Farm household differentiation variables	Net income per capita	income	Annual net household income per capita in 2019	1.1871	1.6359	Negative
	Proportion of non-agricultural labor	non-agri	Ratio of number of households engaged in non-farm labor to total household labor in 2019	0.2360	0.2370	Negative
Household head characteristic variables	Gender	gender	Female = 0, Male = 1	0.9026	0.2966	Negative
	Age	age	Age value of head of household 2019	55.3652	10.7449	Positive
	Health status	health	Health status of the head of household in 2019, non-healthy = 0, healthy = 1	0.7956	0.4034	Negative
	Education level	edu	Years of education for heads of household in 2019	5.9474	4.0937	Negative
Human capital	Household size	size	Total household size in 2019	4.2839	1.7784	Positive
	Population burden coefficient	dep_rate	Ratio of the number of persons in the household not in the labor force to the total number of persons in the household labor force in 2019	0.3662	0.3084	Positive
	Access to professional skills training status	tra_rate	Ratio of the number of workers in the household trained in professional skills to the total number of workers in the household	0.1426	0.2474	Negative
Natural capital	Land area operated per capita (mu)	land	Land area operated by households per capita in 2019 (mu/person)	2.1196	3.2085	Negative
Social capital	Number of relatives in close contact	relative	Number of families who visited each other and were able to provide information sharing or material assistance in 2019 (households)	13.1118	10.0387	Negative
Material capital	Number of productive assets	asset	Number of productive household assets (pieces/units) in 2019	0.9510	1.2364	Negative

^1^ The results of the poverty vulnerability measures for farmers are all between 0 and 1, and all are greater than 0, with the smallest value being infinitely close to 0.

**Table 2:** Model estimation results and robustness tests.

Variable	Variable Name	Equation (1)	Equation (2)	Equation (3)	Equation (4)	Equation (5)
Farm household differentiation variables	Net income per capita	−0.0040 *	−0.0045 **	−0.0051 **	−0.0040 *	−0.0038 **
		(0.0023)	(0.0023)	(0.0022)	(0.0023)	(0.0019)
	Proportion of non-agricultural labor	−0.2495 ***	−0.2157 ***	−0.2644 ***	−0.2467 ***	−0.2265 ***
		(0.0218)	(0.0211)	(0.0223)	(0.0213)	(0.0181)
Household head characteristic variables	Gender	−0.0246 **	−0.0124	−0.0235 **	−0.0242 **	−0.0216 **
		(0.0116)	(0.0114)	(0.0113)	(0.0113)	(0.0096)
	Age	0.0037 ***	0.0033 ***	0.0040 ***	0.0036 ***	0.0027 ***
		(0.0004)	(0.0004)	(0.0004)	(0.0004)	(0.0004)
	Health status	−0.0509 ***	−0.0537 ***	−0.0502 ***	−0.0491 ***	−0.0357 ***
		(0.0082)	(0.0083)	(0.0080)	(0.0081)	(0.0069)
	Education level	0.0015	—	0.0012	0.0015 *	0.0014 *
		(0.0009)		(0.0009)	(0.0009)	(0.0008)
Human capital	Household size	0.0199 ***	—	0.0185 ***	0.0198 ***	0.0191 ***
		(0.0022)		(0.0021)	(0.0021)	(0.0018)
	Population burden coefficient	0.1386 ***	0.1322 ***	0.1399 ***	0.1394 ***	0.1464 ***
		(0.0132)	(0.0132)	(0.0134)	(0.0129)	(0.0108)
	Access to professional skills training status	−0.0954 ***	−0.0878 ***	—	−0.0950 ***	−0.0916 ***
		(0.0195)	(0.0199)		(0.0191)	(0.0162)
Natural capital	Land area operated per capita (mu)	0.0017	−0.0009	—	0.0018	0.0027 **
		(0.0013)	(0.0009)		(0.0013)	(0.0011)
Social capital	Number of relatives in close contact	−0.0020 ***	−0.0017 ***	−0.0022 ***	−0.0019 ***	−0.0017 ***
		(0.0005)	(0.0005)	(0.0005)	(0.0005)	(0.0004)
Material capital	Number of productive assets	0.0161 ***	0.0198 ***	0.0167 ***	0.0161 ***	0.0158 ***
		(0.0031)	(0.0031)	(0.0029)	(0.0030)	(0.0024)
	Constant	0.3649 ***	0.4580 ***	0.3541 ***	0.3620 ***	0.3424 ***
		(0.0291)	(0.0267)	(0.0295)	(0.0285)	(0.0240)
	Observations	1673	1673	1673	1673	1673
	R-squared	0.2489	0.2062	0.2280	0.2528	0.2870

Note: ***, ** and * denote coefficients of explanatory variables significant at 1%, 5% and 10% levels, respectively.

**Table 3:** Contribution of residual, constant, and explanatory variables to poverty vulnerability.

	Coefficient of Variation	Contribution Rate (%)
Total	3.4240	100
Residual term	0.9668	28.24
Constant term	−0.2333	−6.81
Explanatory variables	2.6905	78.58

**Table 4:** Contribution of each explanatory variable to the vulnerability of farm households to poverty.

Variable	Variable Name	Coefficient of Variation	Contribution Rate (%)
Farm household differentiation variables	Net income per capita	0.0402	1.49
	Proportion of non-agricultural labor	0.9430	35.05
Household head characteristic variables	Gender	0.0077	0.29
	Age	0.1757	6.53
	Health status	0.0778	2.89
	Education level	0.0285	1.06
Human capital	Household size	0.2971	11.04
	Population burden coefficient	0.3439	12.78
	Access to professional skills training status	0.3100	11.52
Natural capital	Land area operated per capita (mu)	0.0967	3.59
Social capital	Number of relatives in close contact	0.0968	3.60
Material capital	Number of productive assets	0.2731	10.15
	Total of all variables	2.6905	100.00

## Data Availability

If necessary, we can provide raw data.

## Supplementary Materials

The following supporting information can be downloaded at: https://www.mdpi.com/article/10.3390/ijerph19084878/s1, Table S1: The questionnaire of the living conditions of rural families.
