# Supplementary material for: Rural Household Differentiation and Poverty Vulnerability: An Empirical Analysis Based on the Field Survey in Hubei, China
Source: Int J Environ Res Public Health. 2022 Apr 17;19(8):4878. doi: 10.3390/ijerph19084878 (PMC9031628; doi:10.3390/ijerph19084878)
Supplement: Supplementary file 1 [file ijerph-19-04878-s001.zip › ijerph-1665607-supplementary.pdf]

Farmer's code__________________

Table S1 The questionnaire of the living conditions of rural families

Province			
County			
Township/Town			
Village			
Name of Householder		Corresponding code	
Respondent		Corresponding code	
Economic status（P/N/R）			
Phone number			
Inquirer			
Date and time			

A. Family demographic information (listing all family members, starting with the head of household)

	Personal code	01	02	03	04	05	06	07	08	09	10
01	Relationship with householder (1 householder; 2 a spouse; 3 son; 4 daughter-in-law; 5 daughters; 6 son-in-law; 7 grandchildren/daughter; 8 father/mother; 9 grandfather/grandmother; 10 = others)										
02	Gender (1 male; 2 female)										
03	Age (years)										
04	Ethnic Group (1 Han, 2 Others)										
05	Marital Status (1 single; 2 first marriage; 3 remarried; 4 divorced; 5 widowed)										
06	Education level (1 illiterate; 2 primary school; 3 junior high school; 4 senior high school or technical secondary school; 5 junior college or above)										
07	Professional training (1= yes; 2 = no)										
08	Current political status (1 CPC member, 2 Democratic parties, 3 people)										
09	Current Main occupation (above 16 years old) 1 farming at home; 2 agricultural work; 3 non-agricultural work; 4 self-owned enterprise; 5 village cadre; 6 working in government departments or enterprises or institutions; 7 student; 8 doing nothing; 9 other (give clear indication)										
10	Working place (1 within the township; 2 within the county outside the township; 3 within the province outside the county; 4 within the country outside the province; 5 abroad)										
11	Time of agricultural production activities (month)										
12	Time of working in other places (month)										
13	How healthy are you compared to your peers (1 very poor; 2 relatively poor; 3 fair; 4 fairly good; 5 very good)										
14	Mobility (1 can do normal activities; 2 can't do heavy farm work but can do some light farm work; 3 can't do any farm work but can do some light housework; 4 can't do housework but can take care of oneself; 5 can't take care of life)										
15	Whether the householder has a daughter married (1 yes; 2 no)										

16. Additional description of household demographic information:

B. The natural and physical assets of the household

Type	Quantity	Purchase date	Type	Quantity	Purchase date	Type	Quantity	Purchase date	Land resources	Area (mu)
electric fan			computer			the water pump			farmland	
washing machine			camera			draught animals			woodland	
refrigerator			phone/mobile			1.			aquaculture water area	
air conditioner			production car			2.				
the bicycle			the tractor			3.				
motorcycle /electric car			motor tricycle			livestock products				
life car			tilling machine			1.				
modern stove			harvesting machinery			2.				
TV			threshing machinery			3.				
audio equipment			power machinery							

Note: Purchase year refers to the earliest purchase year; Modern stove refers to the range hood, rice cooker, microwave oven, water heater, gas stove, etc. Power machinery including gasoline engine, diesel engine, internal combustion engine, etc.

01	Total number of houses	
02	Living space (m^2)	
03	The number of floors in a house: 1 bungalow 2 two floors 3 more than two floors	
04	Wall materials: 1 thatch 2 soil and wood 3 brick, stone or cement 4 reinforced concrete	
05	Floor materials: 1 earth 2 brick or stone 3 cement 4 tile or wood	
06	Primary cooking energy: 1 firewood and grass 2 biogas 3 coal 4 gas or natural gas 5 electricity	
07	Toilet type: 1 no toilet 2 pit privy 3 flush toilet	

C. The social and financial capital of the family

01	How many relatives did you visit and visited you during the Spring Festival?	
02	These relatives are engaged in the following jobs:<br>1 government officials 2.the owner of an enterprise 3 doctor 4 teacher 5 others (give clear indication)	
03	If there's a family emergency and you need money (for example, the child gets married), how much can the home take out?<br>1.Less than5000 RMB 2.5000RMB-10000RMB 3.10000RMB-30,000RMB 4.30000RMB-50000RMB<br>5.50,000RMB-100,000 RMB 6.100,000RMB or more	
04	If there's a family emergency and you need money (for example, the child gets married), but the family has no money. How much money can the family raise by borrowing money and taking out loans?<br>1. Less than 5000 RMB 2. 5000RMB-10000RMB 3.10000RMB-30,000RMB 4.30000RMB-50000RMB<br>5.50,000RMB-- 100,000 RMB 6.100,000 or more	

D. Family income

1. Crop income

Variety	Area（mu）	Total output（kg/mu）	Sales（kg）	Price（yuan/kg）	Output value（yuan）	Input（yuan）					
						Seeds	Pesticides Fertilizers	Machinery	Employees	Other	Total

2. Farming income

Variety	The output size（number or kg）	Quantity of sale（number or kg）	Price（yuan/kg）	Sales revenue（yuan）	Input（yuan）				
					Seedlings	Feed	Epidemic prevention	Other	Total

3. Other income

Other operating income	Income（yuan）	Wage income	Income（yuan）	Transfer income	Income（yuan）	Property income	Income（yuan）

Other business: 1 Individual processing 2 Individual small business etc.; Wage income: 1 Agricultural casual laborer 2 Non-agricultural casual laborer 3 Long term working in other places 4 Wages for working in government and public institutions 5 other（give clear indication）

Transfer income: 1 Collective distribution 2 Relief 4 Subsidy 5 Cash gift（the favor pattern） 6 Endowment insurance or pension; Property income: 1＝interest 2＝dividend 3＝the rent 4＝other

E. Family life course

Important events

Income change

Living activities

Changes in assets

Population change

The starting date

In 2019

The changes of population:1 Birth; 2 Get married(man); 3 Get married(woman); 4 Divorce; 5 Death; Other（give clear indication）

The changes of assets:1 To build a house；2 Decoration; 3 Agricultural machinery; 4 White goods; 5 Expropriating land; 6 Buy cars（give clear indication）

Living activities:1 Planting industry structure change; 2 Breeding change; 3 Work outside; 4 Form a company; 5 Other（give clear indication）

The changes of income（the reason of economic hardship）: A A large amount of education spending; B Disease；C The loss of main labor; D Natural disasters; E operating losses; F Arrange weddings and funerals; G Other（give clear indication）

F. Open questions

1、What external policies have had a significant impact on family production and life ? The impact is embodied in what respect?

2、Whether the external environment has a significant impact on family production and life?

3、What do you think is the main reason for the family's financial difficulties over the years? And what makes the economy better?
